# Does maxillary sinus proximity affect molar root resorption during distalization using Invisalign? a CBCT study

**DOI:** 10.1186/s12903-023-03672-x

**Published:** 2023-11-21

**Authors:** Dina Elfouly, Nadia M. El-Harouni, Hanan A. Ismail, Tarek El-Bialy, Ahmed Ghoneima

**Affiliations:** 1 Department of Orthodontics, Faculty of Dentistry, Alexandria University, Champollion St., P.O. Box 21521, Azarita, Alexandria, Egypt; 2 Faculty of Medicine and Dentistry, University of Alberta, Edmonton, Alberta Canada; 3 Hamdan Bin Mohammed College of Dental Medicine, Mohammed Bin Rashid University of Medicine and Health Sciences, Dubai, UAE; 4 Adjunct Faculty, Department of Orthodontics and Oral Facial Genetics, Indiana University School of Dentistry, Indianapolis, IN USA

**Keywords:** Maxillary sinus proximity, Distalization, Clear aligner, Root resorption

## Abstract

**Background:**

This study aimed to assess the correlation between maxillary sinus proximity to root apices of maxillary molars and root resorption during molar distalization using clear aligner therapy (CAT).

**Materials and methods:**

Thirty-eight cone beam computed tomography scans (CBCTs) obtained pre- (T0) and post-treatment (T1) from 19 adult patients (36.68 ± 13.50 years), who underwent maxillary molar distalization using Invisalign® aligners (Align Technology, Inc., San José, CA, USA) with a minimum of 2 mm distalization, were evaluated in this study At least 22 h of aligner wear per day was a main inclusion criterion. Sinus proximity and changes in root lengths were measured for 61 molars (183 roots). Spearman coefficient analysis was used for assessing correlation between sinus proximity and root resorption. The level of significance was set at p ≤ 0.05. The reproducibility of measurements was assessed by intraclass correlation coefficient (ICC).

**Results:**

Spearman coefficient revealed no significant correlation between sinus proximity and molar root resorption for mesiobuccal, distobuccal or palatal roots (p = 0.558, p = 0.334, p = 0.931, respectively).

**Conclusion:**

There was no correlation between maxillary sinus proximity to root apices of maxillary molars and root resorption.

## Introduction

The maxillary sinus floor is structured with compact cortical bone, formed by the alveolar process and part of the hard palate [1]. With age-related pneumatization, the sinus invades the maxillary alveolar process in approximately half of the adult population, coming close to the roots of maxillary second premolars and first and second permanent molars, causing protrusion of the root apices into the sinus [2, 3]. Tooth movement in the cortical bone is an anatomical limitation in adults, due to the difference in bone turnover and surface-active frequency between the cortical and trabecular bones [4, 5].

Sun et al. [6] conducted a systematic review to examine current interventions’ feasibility, safety, and stability for moving teeth through the maxillary sinus. Only nine case reports were included in the review, with only two reports using cone beam computed tomography (CBCT). They demonstrated that the application of constant light to moderate forces to gradually move the teeth through or into the maxillary sinus in adults appears to be feasible and safe. They stated that bodily movement is possible, but teeth seem to be easily tipped initially, potentially resulting in root resorption.

Apical root resorption (ARR), a permanent loss of hard tissue on the root apex of a tooth, is an unavoidable consequence in patients treated using fixed appliances and clear aligner therapy (CAT), although in most cases it is not clinically significant [7, 8]. Severe ARR is uncommon, with an incidence between 1% and 5%, however root resorption can exceed 5 mm or 25% of root length [9].

Since CAT was introduced in the orthodontic practice, it has had a huge impact because of its superior esthetics and comfort [10, 11]. Originally promoted as an alternative treatment for moderate crowding or space closure, aligners are now considered a treatment option for complex cases requiring extraction or distalization [12]. Simon et al. [13] postulated that molar distalization can be accomplished using Invisalign clear aligners system. Additionally, Ravera et al. [14] proposed that 2.25 mm maxillary molar distalization can be accomplished without considerable molar tipping.

However, to date, no study has investigated the possibility that maxillary sinus proximity to the root apices of maxillary molars might affect root resorption during their distalization using CAT. Therefore, the aim of this study was to assess the correlation between maxillary sinus proximity to root apices of maxillary molars and root resorption during molar distalization by CAT using CBCT.

## Materials and methods

This study was approved by the Institutional Review Board at the Faculty of Dentistry, Alexandria University, Alexandria, Egypt (IRB:00010556–IORG:0008839) Manuscript Ethics Committee number (0423-04/2022). All records were of patients who consented for the use of records for research or educational purposes following the ethical approval at University of Alberta, Alberta, Canada protocol # (Pro00091339). All records were de-identified before being enrolled in the study. All methods were carried out in accordance with the Declaration of Helsinki. Informed consent was obtained from all subjects/or their legal guardian(s) for the use of their records. Neither minors nor illiterates were included in this study.

**Fig. 1:**
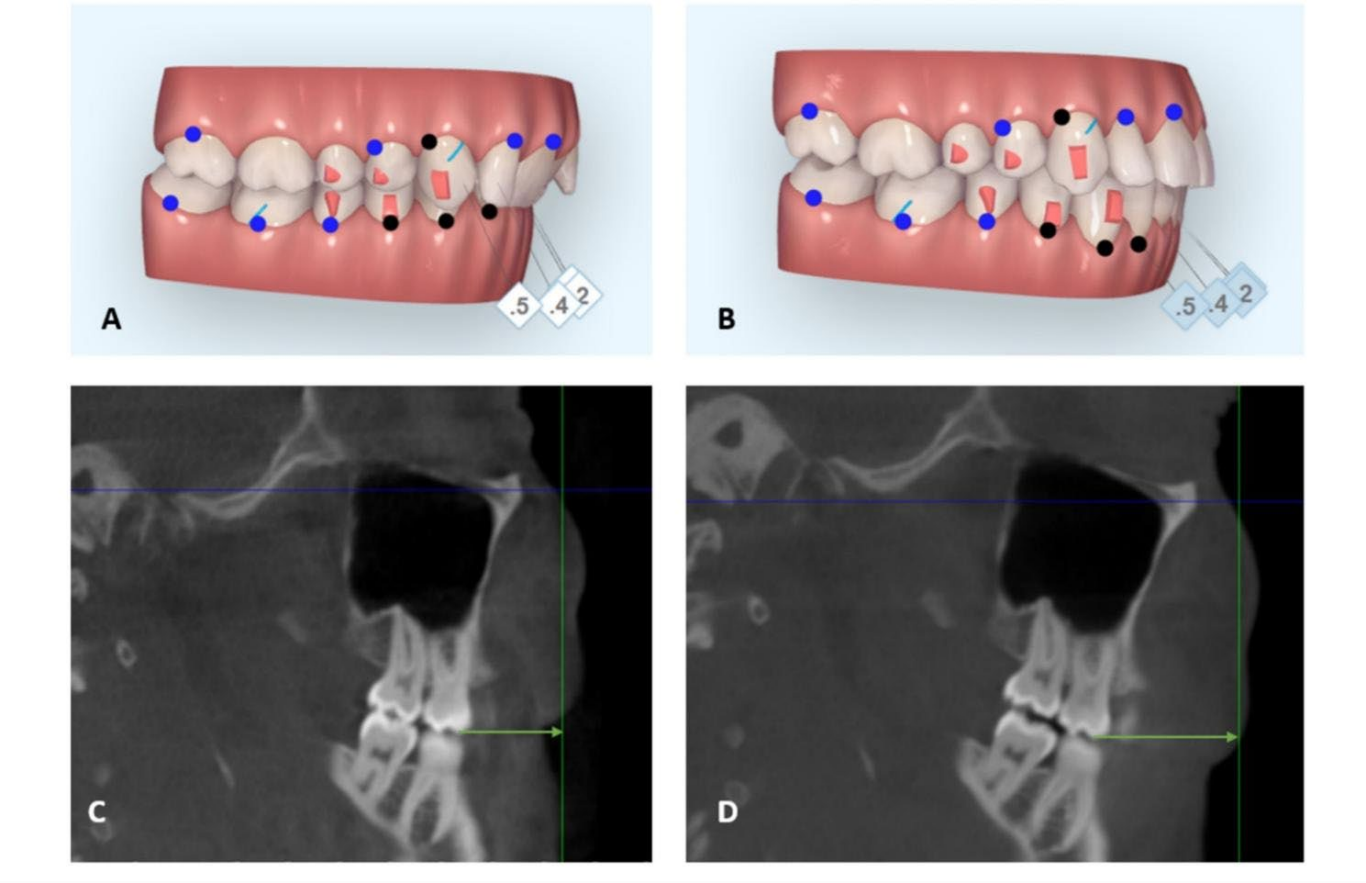
Sequential molar distalization of upper teeth (**A-B**), frames extracted by a ClinCheck (Align Technology, Inc., San Jose, CA, USA). Distance between coronal plane and mesiobuccal cusp of maxillary first molar before (**C**) and after (**D**) distalization

Inclusion criteria: (1) Adult patients with standardized treatment protocol for maxillary sequential molar distalization (Fig. 1, A-B). (2) Patients with a minimum of 2 mm actual molar distalization (as measured on CBCT) (Fig. 1, C-D). (3) Patients selected for the study satisfied the compliance criteria of wearing aligners for at least 22 h per day, as recommended by Invisalign (Align technology, Inc., San Jose, CA, USA) with regular monitoring over six weeks for encouragement. Exclusion criteria: Previous orthodontic treatment and history of systemic disease or craniofacial syndromes or presence of cleft palate.

Thirty-eight cone beam computed tomography scans (CBCTs) obtained pre- (T0) and post-treatment (T1) from 19 adult patients treated with molar distalization using Invisalign by the same health professional in a single center. A total of 61 maxillary molars (183 roots) were included in this study. All CBCT images were taken using the same CBCT machine (i-CAT, Imaging Sciences International (ISI), PA, USA), and the settings used were in accordance with manufacturers’ recommendations (8.9 s, 13 × 16 cm FOV, 120 k, 10 mA and 360° rotation, and voxel size 0.3 mm). All images were acquired with the subjects’ heads positioned such that the Frankfort horizontal plane ran parallel to the floor. Images were saved as Digital Imaging and Communications in Medicine (DICOM) format.

## CBCT image analysis

**Fig. 2:**
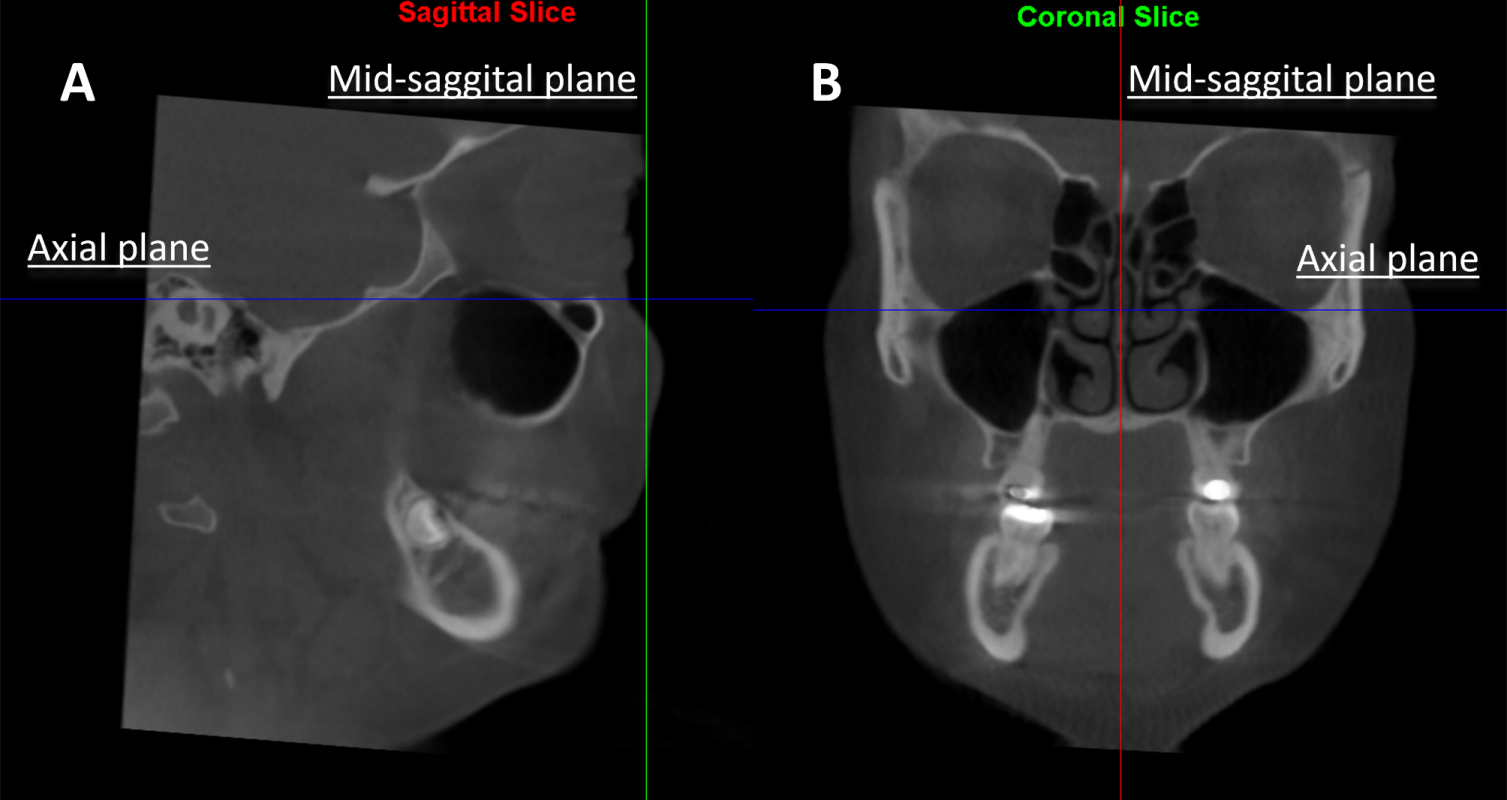
CBCT images were oriented in the sagittal view with coronal plane aligned with Nasion perpendicular and axial plane passing through FHP (**A**) and in the coronal plane with mid-sagittal plane aligned with Nasion perpendicular and axial plane passing through the infraorbital rim (**B**)

Digital CBCT images before and after treatment were evaluated using Dolphin Imaging software v.11.95 Premium (Dolphin Imaging, Chatsworth, CA). Each CBCT image was oriented in the sagittal view with coronal plane aligned with Nasion perpendicular and axial plane passing through the Frankfort horizontal plane (Fig. 2, A). While in the coronal plane, each CBCT image was oriented with mid-sagittal plane aligned with Nasion perpendicular and axial plane passing through the infraorbital rim (Fig. 2, B). Once orientation was carried out, CBCT images and orthogonal planes were not rotated, images were only translated or scrolled, to ensure a reproducible reference plane.

Sagittal images of CBCT were used to measure amount of molar distalization, sinus proximity to root apices and root resorption. Two sagittal sections were used, one showing the mesiobuccal (MB) and distobuccal (DB) root apices, and another showing the palatal (Pa) root apex. Measurements were performed by the same investigator using the measurement tool in Dolphin Imaging software.

## Dental characteristics measurements

### Maxillary molar distalization and maxillary sinus proximity

The amount of maxillary molar distalization was measured using CBCT-driven images in the sagittal plane, linear measurements were taken from the mesiobuccal cusp of the maxillary first molar and mesiobuccal cusp of the maxillary second molar to the coronal plane at (T0) and (T1) (respectively) (Fig. 1, C-D).

**Fig. 3:**
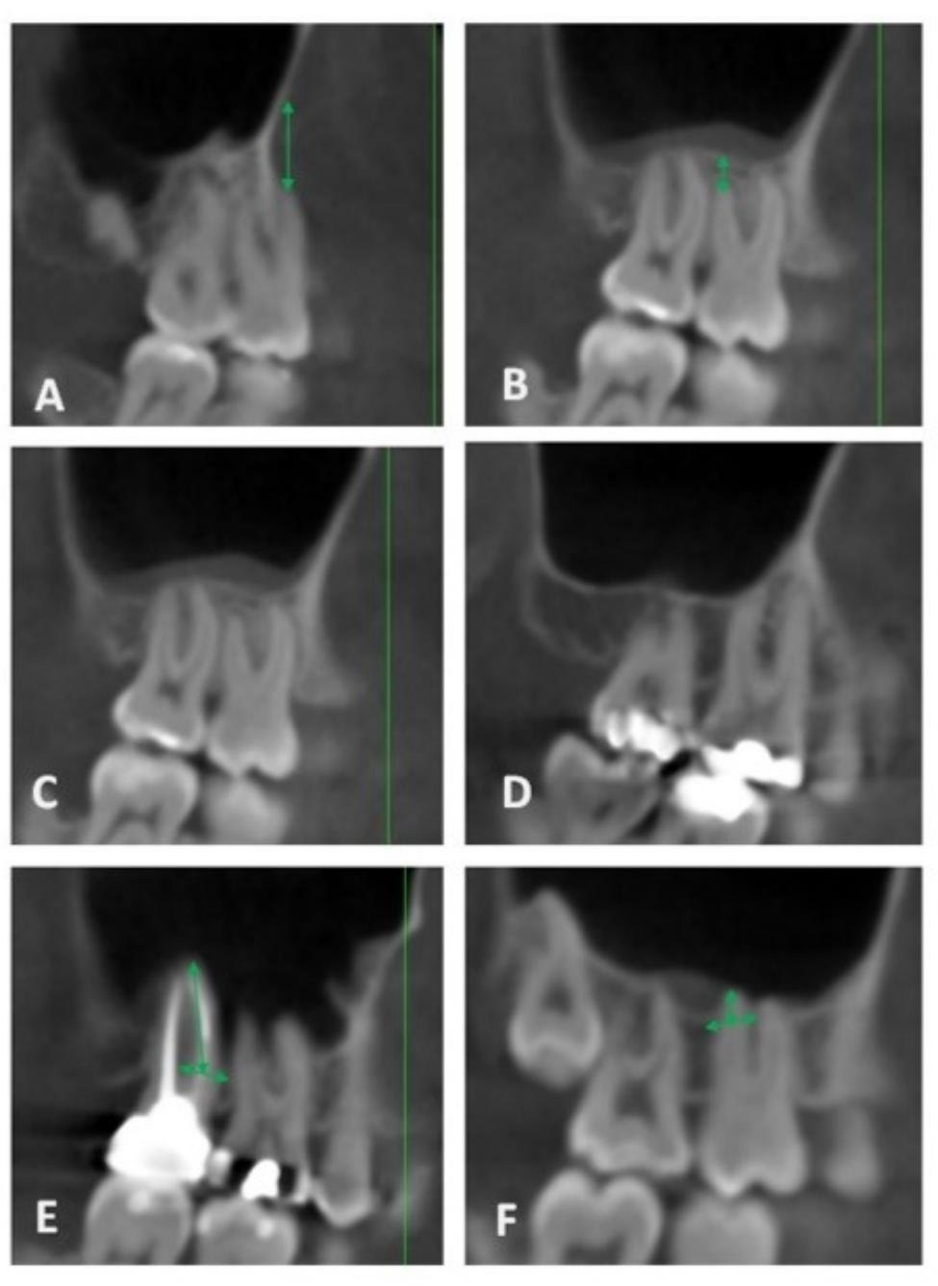
ASD, apex-sinus distance; Type I (**A,B**), Type II (**C,D**) and Type III (**E,F**)

The apex-sinus distance (ASD) – the distance between the root apex of the maxillary molar and the inferior wall of the maxillary sinus – was measured in the sagittal images. Measurements were taken according to the following types: Type I: if the roots had no contact with the inferior wall of the maxillary sinus, the shortest perpendicular distance from the root apex to the inferior wall of the sinus was measured and regarded as a negative value. Type II: if there was contact between the root and the inferior wall, the distance was regarded zero. Type III: if the root protruded into the maxillary sinus, the distance from the root apex to the midpoint between the adjacent contact points of the root and the sinus floor was measured (positive value) [15]. This was repeated for the MB, DB and Pa roots of the maxillary molars (Fig. 3).

### Root length

**Fig. 4:**
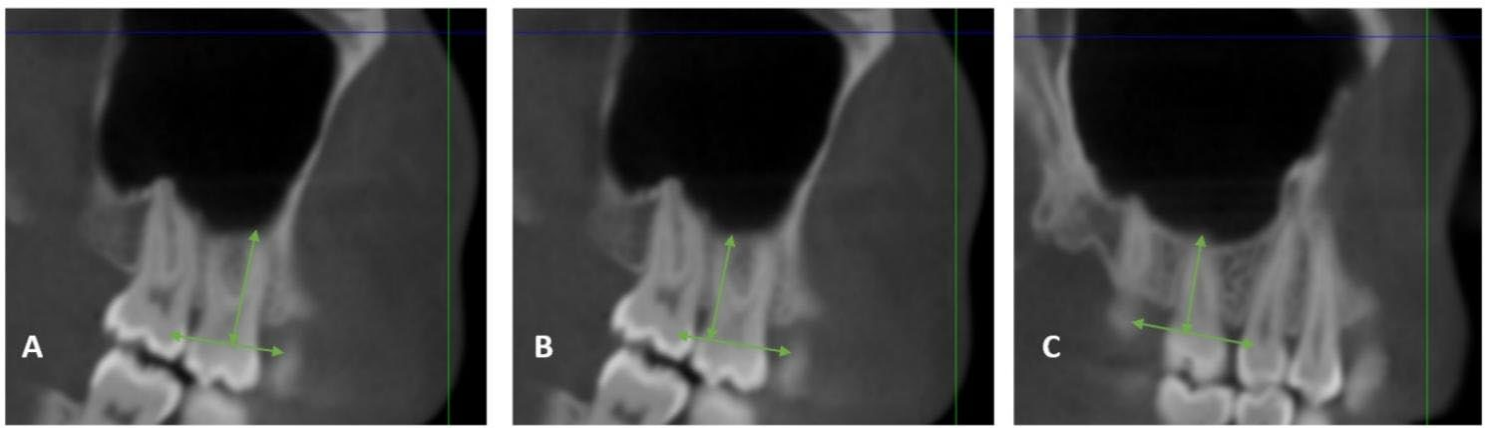
Measurement of root length from CEJ to root apices of the maxillary first permanent molar on sagittal view; (**A**) DB root length, (**B**) MB root length and (**C**) Pa root length

To measure root length, images were scrolled until the best view of the cemento-enamel junction and root apex were displayed simultaneously to ensure accurate measurements of root length. Root length was measured as the perpendicular distance from each root apex to the cemento-enamel junction (CEJ). Cemento-enamel junction was defined as a line joining the mesial and distal cementoenamel junctions of the molar (Fig. 4). This was repeated for the MB, DB, and Pa roots of the first and second maxillary molars, before and after treatment with Invisalign. Root resorption was measured as change in root length before and after treatment.

### Statistical analysis

Prior to conducting the study, ten CBCTs were used to calibrate the investigator. The measurements were repeatedly conducted until an acceptable level of agreement was achieved. All parameters were measured twice by the same examiner one week apart, to assess intra-rater repeatability. Also, a second examiner remeasured ten random cases for assessing inter-rater reliability. This was evaluated using summary statistics for the differences between the repeated measurements and intraclass correlation coefficients (ICCs). Qualitative data were described using numbers and percentages. Quantitative data were described using range (minimum and maximum), mean, and standard deviation. The Kolmogorov-Smirnov and Shapiro-Wilk tests were used to verify the normality of distribution. Spearman coefficient was used to correlate the maxillary sinus proximity and the change in the pre- and post-treatment root lengths with p ≤ 0.05 indicating statistical significance.

## Results

The ICC for intra- rater reliability showed excellent reproducibility for linear measurements (0.94–0.99), in addition to excellent inter-rater reliability (above 0.9) for all measurements [16]. Patients’ demographics; mean age of the patients at start of treatment and sex are presented in (Table 1). The included roots comprised the following percentages: 57% were penetrating the sinus, 27% were in contact with the sinus and 21% were away from sinus. Values for means, SDs, minimum and maximum measurements, and changes between T0 and T1 are reported in (Table 2). Spearman coefficient revealed no significant correlation between sinus proximity and molar root resorption for mesiobuccal, distobuccal or palatal roots (p = 0.558, p = 0.334, p = 0.931, respectively) (Table 3).

**Table 1:** Distribution of the studied cases according to demographic data (n = 19)

	No.	%
**Sex**		
Male	6	31.6
Female	13	68.4
**Age at start of treatment (years)**		
Minimum – Maximum	21.0–62.0	
Mean ± **SD**.	36.68 ± 13.50	

SD: Standard deviation

**Table 2:** Descriptive analysis of the studied cases according to maxillary molars

	n	Min. – Max.	Mean ± SD.
Maxillary Sinus Proximity (mm)			
**MB** root	61	-7.60–10.50	1.02 ± 2.90
**DB** root	61	-2.90–10.50	1.22 ± 2.26
**Pa** root	61	-6.20–8.50	1.06 ± 3.36
Average (all roots)	183	-4.20–8.80	1.10 ± 2.48
**MB** Root length (mm)			
Initial	61	9.20–19.30	13.23 ± 1.60
Final	61	9.30–18.50	12.80 ± 1.60
Decrease	61	-1.0–2.40	0.43 ± 0.69
**DB** Root length (mm)			
Initial	61	6.50–19.30	13.05 ± 1.88
Final	61	6.50–18.50	12.56 ± 1.80
Decrease	61	-0.20–3.0	0.50 ± 0.96
** Pa** Root length (mm)			
Initial	61	9.10–19.30	13.82 ± 1.99
Final	61	8.50–18.10	13.0 ± 1.97
Decrease	61	-0.80–3.50	0.82 ± 0.87

SD: Standard deviation, MB: Mesiobuccal, DB: Distobuccal, Pa: Palatal

**Table 3:** Correlation between maxillary sinus proximity and root apices (mm) and root resorption (mm)

	n	r_s_	p
**Sinus (mm) vs. decrease in root length (mm)**			
**MB** roots	61	-0.076	0.558
**DB** roots	61	0.126	0.334
** Pa** roots	61	0.011	0.931
Average (all roots)	183	-0.007	0.930

Statistically significant at p ≤ 0.05

r_s_: Spearman coefficient, MB: Mesiobuccal, DB: Distobuccal, Pa: Palatal

## Discussion

Resolving Class II molar relationships by distalizing maxillary molars is frequently required in Class II non-extraction patients with minor skeletal discrepancies [17]. Today, sequential molar distalization can be carried out efficiently using aligners when a mean of 2.25–2.6 mm molar distalization movement is required [14, 18].

Previous reports have demonstrated the relationships between the roots of maxillary teeth and the maxillary sinus floor using CBCT [19–25]. CBCT, unlike 2D radiographs, provides precise images of the bone around root apices without distortion or overlapping of surrounding structures [26, 27].

The availability of pre- and post-treatment CBCT records in the current study allowed measurement of sinus proximity to root apices and the amount of root resorption with high specificity, accuracy and reliability [28–31]. Root resorption has not been sufficiently studied in the literature with CAT as Clincheck analysis of three dimensional (3D) models- with no roots represented - is the customary tool used with Invisalign. Moreover, treatment outcome in orthodontics is generally evaluated using 2D) analysis. However, it is difficult to evaluate root resorption accurately by 2D analysis because ARR is a three-dimensional topographical change and overlapping of roots is inevitable [19, 32, 33]. Panoramic and periapical radiographs used to evaluate external apical root resorption (EARR), may cause distortion, thus overestimate or underestimate the extent of resorption. In addition, during orthodontic treatment, angulations of the teeth usually change; thus, the severity of EARR cannot be evaluated accurately with periapical radiography. In recent studies, CBCT overcame these shortcomings and improved the accuracy in measuring root length [28–30, 34].

Correlation analysis showed that sinus proximity to root apices of maxillary molars had no significant correlation with root resorption during distalization using CAT. This may be explained by the nature of the cancellous bone of the maxilla and the maxillary sinus space which do not elicit significant resistance to root movement. The only area of the root that is exposed to cortical bone would be anywhere along the root length, and rarely if ever at the root tip putting into consideration that CAT deliver light forces. Weltman [35] emphasized the importance of the amount and nature of orthodontic forces on root resorption. The nature of forces in CAT itself, which is basically a removable appliance, generates light and discontinuous forces probably explaining the non-significant root resorption [36].

Moreover, root resorption is significantly affected by gender, malocclusion type, crowding, post-treatment approximation to the labial and/or palatal plates [37], amount of overjet before treatment [38], orthodontic treatment with maxillary premolar extraction [38, 39], and duration of active treatment [39–41]. Most of these factors were taken into consideration on designing this study; patients chosen were adult cases with standardized treatment protocol for maxillary sequential molar distalization of a minimum of 2 mm actual molar distalization and were non extraction cases. Furthermore, because the study was focused on molar root resorption during molar distalization, other factors affecting root resorption, like crowding and the amount of overjet, possibly affecting incisors, did not present confounding factors. Still some limitations remained which include variable treatment duration due to patients’ compliance, which had a mean value of 27 months (18 months to 40 months), also the sample was not distributed into groups according to sinus proximity. Moreover, the present study did not consider the type of molar movement i.e., the amount of bodily/ tipping movement of each molar for both crown and root.

Several studies agree with the current results, where Kravitz et al. [42] reported a case of successful maxillary molar intrusion into the maxillary sinus floor without radiographically detectable apical root resorption. Cacciafesta and Melsen [43] reported a case report with maxillary molar distalization without radiographically detectable root resorption on panoramic x-ray. It is worth noting that all previous studies were case reports.

Despite the minimal root resorption observed in all studied molars’ roots (< 1 mm), the amount of root resorption was not clinically significant according to Sharpe,[44] who categorized root resorption of 1–2 mm as 1° severity (mild root resorption or slight blunting of root apex). Li et al. [45] and Aman et al. [36] measured root resorption of anterior teeth during CAT and reached similar results to those of the current study (less than 1 mm resorption). According to Li et al. [45], the apical root resorption of patients treated with aligners was less than that of patients treated with fixed appliances.

## Conclusion

Maxillary sinus proximity has no significant correlation with maxillary molar root resorption during molar distalization using CAT. Within the limitation of this study, maxillary molar root resorption during distalization with CAT may not be clinically significant.

## Data Availability

The datasets used and/or analyzed during the current study are available from the corresponding author on reasonable request.

## Abbreviations

CAT: Clear aligner therapy
2D: two-dimensional
3D CBCT: Three-dimensional cone beam computed tomography
MB: Mesiobuccal
DB: Distobuccal
Pa: Palatal
EARR: External apical root resorption
ASD: Apex-sinus distance
FHP: Frankfurt horizontal plane
CEJ: Cemento-enamel junction
ARR: Apical root resorption

## References

[1] McGrowan D, Baxter P, James J (1993). The maxillary sinus and its dental implications.

[2] Ariji Y, Kuroki T, Moriguchi S, Ariji E, Kanda S (1994). Age changes in the volume of the human maxillary sinus: a study using computed tomography. Dentomaxillofac Radiol.

[3] Lorkiewicz-Muszyńska D, Kociemba W, Rewekant A, Sroka A, Jończyk-Potoczna K, Patelska-Banaszewska M (2015). Development of the maxillary sinus from birth to age 18. Postnatal growth pattern. Int J Pediatr Otorhinolaryngol.

[4] Verna C, Melsen B, Melsen F (1999). Differences in static cortical bone re- modeling parameters in human mandible and iliac crest. Bone.

[5] Birte M (2012). Adult orthodontics.

[6] Sun W, Xia K, Huang X, Cen X, Liu Q, Liu J (2018). Knowledge of orthodontic tooth movement through the maxillary sinus: a systematic review. BMC Oral Health.

[7] Gay G, Ravera S, Castroflorio T, Garino F, Rossini G, Parrini S (2017). Root resorption during orthodontic treatment with Invisalign®: a radiometric study. Prog Orthod.

[8] Villaman-Santacruz H, Torres-Rosas R, Acevedo-Mascarúa AE, Argueta-Figueroa L (2022). Root resorption factors associated with orthodontic treatment with fixed appliances: a systematic review and meta-analysis. Dent Med Probl.

[9] Brezniak N, Wasserstein A (2002). Orthodontically induced inflammatory root resorption. Part I: the basic science aspects. Angle Orthod.

[10] Walton DK, Fields HW, Johnston WM, Rosenstiel SF, Firestone AR, Christensen JC. Orthodontic appliance preferences of children and adolescents. Am J Orthod Dentofacial Orthop. 2010;138:698.e1-12; discussion, 698-9. 10.1016/j.ajodo.2010.06.012 21130314

[11] Fujiyama K, Honjo T, Suzuki M, Matsuoka S, Deguchi T (2014). Analysis of pain level in cases treated with Invisalign aligner: comparison with fixed edgewise appliance therapy. Prog Orthod.

[12] Womack WR (2006). Four-premolar extraction treatment with Invisalign. J Clin Orthod.

[13] Simon M, Keilig L, Schwarze J, Jung BA, Bourauel C (2014). Treatment outcome and efficacy of an aligner technique–regarding incisor torque, premolar derotation and molar distalization. BMC Oral Health.

[14] Ravera S, Castroflorio T, Garino F, Daher S, Cugliari G, Deregibus A (2016). Maxillary molar distalization with aligners in adult patients: a multicenter retrospective study. Prog Orthod.

[15] Oishi S, Ishida Y, Matsumura T, Kita S, Sakaguchi-Kuma T, Imamura T (2020). A cone-beam computed tomographic assessment of the proximity of the maxillary canine and posterior teeth to the maxillary sinus floor: lessons from 4778 roots. Am J Orthod Dentofacial Orthop.

[16] Bartko JJ (1966). The intraclass correlation coefficient as a measure if reliability. Psychol Rep.

[17] Bolla E, Muratore F, Carano A, Bowman SJ (2002). Evaluation of maxillary molardistalization with the distal jet: a comparison with other contemporary methods. Angle Orthod.

[18] Saif BS, Pan F, Mou Q, Han M, Bu W, Zhao J (2022). Efficiency evaluation of maxillary molar distalization using Invisalign based on palatal rugae registration. Am J Orthod Dentofacial Orthop.

[19] Sharan A, Madjar D (2006). Correlation between maxillary sinus floor topography and related root position of posterior teeth using panoramic and cross-sectional computed tomography imaging. Oral Surg Oral Med Oral Pathol Oral Radiol Endod.

[20] Kwak HH, Park HD, Yoon HR, Kang MK, Koh KS, Kim HJ (2004). Topographic anatomy of the inferior wall of the maxillary sinus in koreans. Int J Oral Maxillofac Surg.

[21] Jung YH, Cho BH (2012). Assessment of the relationship between the maxillary molars and adjacent structures using cone beam computed tomography. Imaging Sci Dent.

[22] Pagin O, Centurion BS, Rubira-Bullen IR, Alvares Capelozza AL (2013). Maxillary sinus and posterior teeth: accessing close relationship by cone-beam computed tomographic scanning in a Brazilian population. J Endod.

[23] Kang SH, Kim BS, Kim Y (2015). Proximity of posterior teeth to the maxillary sinus and buccal bone thickness: a biometric assessment using cone-beam computed tomography. J Endod.

[24] Estrela C, Nunes CA, Guedes OA, Alencar AH, Estrela CR, Silva RG (2016). Study of anatomical relationship between posterior teeth and maxillary sinus floor in a subpopulation of the Brazilian central region using cone-beam computed tomography—part 2. Braz Dent J.

[25] Ahn NL, Park HS (2017). Differences in distances between maxillary posterior root apices and the sinus floor according to skeletal pattern. Am J Orthod Dentofacial Orthop.

[26] American Academy of Oral and Maxillofacial Radiology (2013). Clinical recommendations regarding use of cone beam computed tomography in orthodontics. Position statement by the American Academy of Oral and maxillofacial Radiology. Oral Surg Oral Med Oral Pathol Oral Radiol.

[27] Hodges RJ, Atchison KA, White SC (2013). Impact of cone-beam computed tomography on orthodontic diagnosis and treatment planning. Am J Orthod Dentofacial Orthop.

[28] Ren H, Chun J, Deng F, Zheng L, Liu X, Dong Y (2013). Comparison of cone-beam computed tomography and periapical radiography for detecting simulated apical root resorption. Angle Orthod.

[29] Patel S, Dawood A, Wilson R, Horner K, Mannocci F (2009). The detection and management of root resorption lesions using intraoral radiography and cone beam computed tomography—an in vivo investigation. Int Endod J.

[30] Estrela C, Bueno MR, De Alencar AH, Mattar R, Valladares Neto J, AzevedoBC (2009). Method to evaluate inflammatory root resorption by using cone beam computed tomography. J Endod.

[31] Samandara A, Papageorgiou SN, Ioannidou-Marathiotou I, Kavvadia-Tsatala S, Papadopoulos MA (2019). Evaluation of orthodontically induced external root resorption following orthodontic treatment using cone beam computed tomography (CBCT): a systematic review and meta-analysis. Eur J Orthod.

[32] Wehrbein H, Fuhrmann RA, Diedrich PR (1995). Human histologic tissue response after long-term orthodontic tooth movement. Am J Orthod Dentofacial Orthop.

[33] Park JH, Tai K, Kanao A, Takagi M (2014). Space closure in the maxillary posterior area through the maxillary sinus. Am J Orthod Dentofacial Orthop.

[34] Dudic A, Giannopoulou C, Martinez M, Montet X, Kiliaridis S (2008). Diagnostic accuracy of digitized periapical radiographs validated against micro-computed tomography scanning in evaluating orthodontically induced apical root resorption. Eur J Oral Sci.

[35] Aman C, Azevedo B, Bednar E, Chandiramami S, German D, Nicholson E (2018). Apical root resorption during orthodontic treatment with clear aligners: a retrospective study using cone-beam computed tomography. Am J Orthod Dentofacial Orthop.

[36] Fernandes LQ, Figueiredo NC, Antonucci CC, Lages EM, Andrade I, Junior JC (2019). Predisposing factors for external apical root resorption associated with orthodontic treatment. Korean J Orthod.

[37] Pastro JD, Nogueira AC, de Freitas KM, Valarelli FP, Cançado RH, de Oliveira RC (2018). Factors associated to apical root resorption after orthodontic treatment. Open Dent J.

[38] Yassir YA, McIntyre GT, Bearn DR (2021). Orthodontic treatment and root resorption: an overview of systematic reviews. Eur J Orthod.

[39] Yi J, Xiao J, Li Y, Li X, Zhao Z (2018). External apical root resorption in non-extraction cases after clear aligner therapy or fixed orthodontic treatment. J Dent Sci.

[40] Weltman B, Vig KW, Fields HW, Shanker S, Kaizar EE (2010). Root resorption associated with tooth movement: a systematic review. Am J Orthod Dentofacial Orthop.

[41] Sawicka M, Bedini R, Wierzbicki PM, Pameijer CH (2015). Interrupted or-thodontic force results in less root resorption than continuous force in human premolars as measured by microcomputed tomography. Folia Histochem Cytobiol.

[42] Kravitz ND, Kusnoto B, Tsay PT, Hohlt WF (2007). Intrusion of overerupted upper first molar using two orthodontic microscrews. Angle Orthod.

[43] Cacciafesta V, Melson B (2001). Mesial bodily movement of maxillary and mandibular molars with segmented mechanics. Clin Orthod Res.

[44] Sharpe W, Reed B, Subtelny JD, Polson A (1987). Orthodontic relapse, apical root resorption, and crestal alveolar bone levels. Am J Orthod Dentofacial Orthop.

[45] Li Y, Deng S, Mei L, Li Z, Zhang X, Yang C (2020). Prevalence and severity of apical root resorption during orthodontic treatment with clear aligners and fixed appliances: a cone beam computed tomography study. Prog Orthod.

